# The c.1460C>T Polymorphism of *MAO-A* Is Associated with the Risk of Depression in Postmenopausal Women

**DOI:** 10.1100/2012/194845

**Published:** 2012-04-24

**Authors:** R. Słopień, A. Słopień, A. Różycka, A. Warenik-Szymankiewicz, M. Lianeri, P. P. Jagodziński

**Affiliations:** ^1^Department of Gynecological Endocrinology, Poznan University of Medical Sciences, Ul. Polna 33, 60-535 Poznan, Poland; ^2^Department of Child and Adolescent Psychiatry, Poznan University of Medical Sciences, Ul. Szpitalna 27/33, 60-572 Poznan, Poland; ^3^Department of Biochemistry and Molecular Biology, Poznan University of Medical Sciences, Ul. Święcickiego 6, 60-781 Poznan, Poland

## Abstract

*Objective*. The aim of the study was an evaluation of possible relationships between polymorphisms of serotoninergic system genes and the risk of depression in postmenopausal women. *Methods*. We studied 332 women admitted to our department because of climacteric symptoms. The study group included 113 women with a diagnosis of depressive disorder according to the Hamilton rating scale for depression; the controls consisted of 219 women without depression. Serum 17*β*-estradiol concentrations were evaluated using radioimmunoassay, while polymorphisms in serotoninergic system genes: serotonin receptors 2A (*HTR2A*), 1B (*HTR1B*), and 2C (*HTR2C*); tryptophan hydroxylase 1 (*TPH1*) and 2 (*TPH2*), and monoamine oxidase A (*MAO-A*) were evaluated using polymerase chain reaction-restriction. *Results*. We found that the 1460T allele of *MAO-A* c.1460C>T (SNP 1137070) appeared with a significantly higher frequency in depressed female patients than in the control group (*P* = 0.011) and the combined c.1460CT + TT genotypes were associated with a higher risk of depression (*P* = 0.0198). Patients with the 1460TT genotype had a significantly higher 17*β*-estradiol concentration than patients with the 1460CT genotype (*P* = 0.0065) and 1460CC genotype (*P* = 0.0018). *Conclusions*. We concluded that depression in postmenopausal women is closely related to the genetic contribution of *MAO-A*.

## 1. Introduction

**Figure 1:**
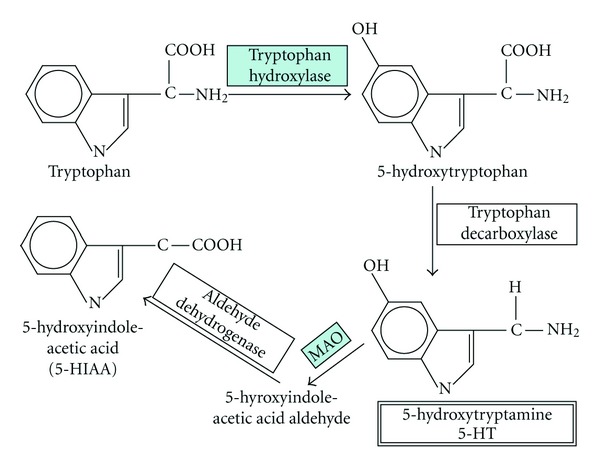
Contribution of tryptophan hydroxylase and MAO in serotonin metabolism. Tryptophan hydroxylase catalyzes the monooxygenation of tryptophan to 5-hydroxytryptophan, which is subsequently decarboxylated to form serotonin (5-hydroxytryptamine; 5-HT). Monoamine oxidase (MAO) catalyzes the oxidative deamination of 5-HT to the corresponding aldehyde. This is followed by oxidation by aldehyde dehydrogenase to 5-HIAA, the indole acetic acid derivative.

The central serotoninergic system has been implicated in the pathophysiology of a number of neuropsychiatric disorders such as mood disorders, substance abuse, or alcoholism [1, 2]. Well-studied components of this system are the serotonin (5-HT) receptors 2A (5-HT2A), 1B (5-HT1B), and 2C (5-HT2C), and the key regulators of 5-HT metabolism: tryptophan hydroxylase 1 and 2 (TPH1 and TPH2) and monoamine oxidase A (*MAO-A*) (Figure 1). The receptors 5-HT2A, 5-HT2C, and 5-HT1B are members of a family of receptors linked to guanine-nucleotide-binding proteins (G-protein-coupled receptor; GPCR) expressed on the cell body and dendrites of serotoninergic neurons in the brain [3]. The receptors 5-HT2A and 5-HT2C are the main excitatory receptor subtypes among the GPCRs for 5-HT, whereas the 5-HT1B receptor is thought to act as a nerve terminal autoreceptor, inhibiting the release of 5-HT. After acting at its receptor, 5-HT is metabolized by *MAO-A* (Figure 1), and therefore MAO activity may play a critical role in the regulation of the serotoninergic system and in the pathogenesis of depressive disorders [4].

The gene encoding 5-HT2A (*HTR2A*) is considered to be a candidate gene for depression. Genetic association has been reported between the c.102C>T polymorphism in the *HTR2A* gene and depression, as well as suicidal behavior in patients with mood disorders and schizophrenia [5–7], although several studies have failed to replicate these findings [8]. It is likely that other serotoninergic genes are involved in gene-environment interactions related to depression. Among them, the gene encoding the 5-HT1B receptor (*HTR1B*) has been correlated to attempted suicide in patients with major depression, because altered postmortem 5-HT1B receptor binding was found to be associated with suicide in some of the studies [9]. A common c.861 G>C polymorphism of the *HTR1B *gene was identified in the coding region of the gene, and major depression appears to be associated with this *locus *[10]. A strong association between suicide and receptor genes of the serotoninergic system has presented evidence for yet another 5-HT receptor gene located on human chromosome Xq24, the 5-HT2C receptor gene (*HTR2C*) [11]. This receptor mediates the release of dopamine (DA) in the brain and can cause anxiety, depression, and compulsive behaviors in human subjects due to the mechanism of rapid downregulation by serotonin. The structural variant c.68 G>C of the* HTR2C* gene that gives rise to a cysteine-to-serine substitution in the *N* terminal extracellular domain of the receptor protein (Cys23Ser) has recently been found to be significantly associated with female suicide victims [12].

Tryptophan hydroxylase (TPH) is the rate-limiting enzyme in the biosynthesis of 5-HT and thus has a major function in regulating the serotoninergic system [13]. Studies of the human brain have revealed two isoforms of the gene coding for tryptophan hydroxylase 1 and 2, termed *TPH1 *and *TPH2.* One of them, the *TPH2 *gene, is of interest because this tryptophan hydroxylase 2 isoform regulates the biosynthetic pathway of 5-HT in the serotoninergic neurons of *raphe nuclei* and has been implicated in the pathogenesis of major depressive disorder and the mechanism of antidepressant action [14].

Investigations of the functional effects of genetic variations in the *TPH2 *gene have demonstrated that a haplotype of 3 SNPs within the gene promoter (−703G>T, −473T>A, and 90 A>G) influenced transcription in human cell lines. Similarly, the *TPH2 *c.1077G>A polymorphism within the coding region (Pro312Pro; SNP 7305115) increased expression levels of the gene in the human pons, containing the dorsal and median *raphe nuclei *[15]. This SNP has previously demonstrated an association with major depression and suicide [16].

Monoamine oxidase (MAO) is an important enzyme associated with the metabolism of biogenic amines and neurotransmitters, including norepinephrine (NE), DA, and 5-HT. Two forms of the enzyme, MAOA and MAOB, are found in the human brain [17]. Of the two, MAOA exhibits a higher affinity for 5-HT and NE, whereas DA is preferentially metabolized by both forms of the enzyme. The MAOA gene (*MAO-A*) has been mapped to the short arm of the *X* chromosome; thus, functional polymorphisms of this *locus* are expected to manifest in a sex-specific fashion. The human *MAO-A* contains a variable-number tandem repeat (VNTR) polymorphism in its promoter region that may alter the transcriptional efficiency of MAOA expression [18]. Most of the other known *MAO-A *polymorphisms either affect intronic sequences or introduce a silent change in the open-reading frame (i.e., the *Eco*RV polymorphism and* Fnu*4HI polymorphism, SNPs: 1137070 and 6323, resp.). These variants are unlikely to affect MAO function, although they may be in disequilibrium with other, as yet unidentified, functional variants. The primary role of MAOA in regulating monoamine turnover, and hence ultimately influencing levels of NE, DA, and 5-HT, indicates that its gene is a highly plausible candidate for affecting individual differences in the manifestation of psychological traits and psychiatric disorders [19, 20]. For example, several studies indicate that the *MAO-A* gene may be involved in the pathogenesis of depression and major depressive disorder [21, 22].

However, genetic factors can modulate the risk for depression by influencing monoaminergic activity in a sexually dimorphic manner. Because the *MAO-A* gene is X-linked, males are hemizygous at this *locus*, whereas females are homozygous or heterozygous. Moreover, the *MAO-A Eco*RV polymorphism was found to be associated with depression in males but not in females [23, 24]. A sexually dimorphic pattern of genetic susceptibility to obsessive-compulsive disorder (OCD) may also be present [25–27]. Statistically significant associations were observed between the alleles for the *MAO-A Eco*RV polymorphism and levels of MAO activity in human male fibroblast lines [28]. The functional analyses of this genetic variation have revealed the existence of the high-activity T allele and the low-activity C allele of the *MAO-A Eco*RV polymorphism.

Individuals with a high-activity *MAO-A* genotype would be expected to have greater serotonin turnover (Figure 1). High plasma MAO activity, however, has been found to be significantly correlated with testosterone levels in men and with 17*β*-estradiol levels in women, with high testosterone or 17*β*-estradiol levels leading to low plasma MAO activity [29]. Women are more likely than men to develop affective disorders, including depression, and this risk increases after menopause, when estrogen production in the ovaries ends [30–33]. We hypothesized that the high-activity *MAO-A* genotype would be associated with depression in postmenopausal women. To test this hypothesis, we evaluated serum 17*β*-estradiol concentrations and genotyped the *MAO-A Eco*RV polymorphism in a group of healthy (*n* = 219) and depressed (*n* = 113) postmenopausal women. We also used a case-control study design in the same individuals to investigate a possible association with a susceptibility to depression of the SNPs of five other serotoninergic system genes: *5HTR2A* (SNP 6313), *5HTR1B* (SNP 6296), *HTR2C *(SNP6318),* TPH1 *(SNP 1800532), and *TPH2* (SNP 7305115).

## 2. Patients and Methods

### 2.1. Patients

We studied three hundred and thirty-two postmenopausal women, aged 42–67, who were admitted to the Department of Gynecological Endocrinology, Poznan University of Medical Sciences, because of climacteric complaints. All postmenopausal women had their last menstrual flow more than 1 year before the study.

All patients were assessed with the Hamilton rating scale for depression (HRSD) and divided into two groups: diagnosed of depressive disorder (113 women) and without depression (219 women served as the control group). According to HRSD, mild depression was defined as a score more than 7 and less or equal to 17, and moderate depression was defined as a score more than 17 and less or equal to 25. The 113 women with depression included 82 women with mild depression and 31 women with moderate depression. None of the examined women were on hormone replacement therapy (HRT) or on psychotropic drugs.

All experiments were carried out after obtaining informed consent from all the participating women. The local Ethic Review Committee of Poznan University of Medical Sciences approved the study protocol.

### 2.2. Blood Samples and Measurements of 17*β*-Estradiol

A blood sample was collected from each study participant. 17*β*-estradiol serum concentrations were quantified using radioimmunoassay (RIAs). The intraassay and interassay coefficients of variation (CV) were 1.2–3.3%, and 2.0–5.6%, respectively.

### 2.3. Genotyping by RFLP

Genomic DNA was prepared from sodium-versenate-(EDTANa_2_-) treated blood samples. Genotyping for polymorphisms in *5HTR2A* c.102C>T (SNP 6313), *5HTR1B* c.861G>C (SNP 6296), *HTR2C *c.68G>C (SNP6318),* MAO-A* c.1460C>T (SNP 1137070),* TPH1 *218C>A (SNP 1800532), and *TPH2* c.1077A>G (SNP 7305115) was determined by polymerase chain reaction-restriction fragment length polymorphism (PCR-RFLP) assay, using the appropriate restriction enzymes. The digested PCR products were resolved on a 2% agarose gel and stained with ethidium bromide for visualization under UV light.

### 2.4. Statistical Analysis

The genotype and allele frequencies of all analyzed polymorphisms were compared between the group of postmenopausal women with depression and without depression using a case-control study design. Significance was evaluated by the Fisher exact test. Odds ratios (ORs) and 95% confidence intervals (CIs) were estimated using the GraphPad (Instant, USA) program. An online (http://ihg2.helmholtz-muenchen.de/cgi-bin/hw/hwa1.pl) program for deviation from the Hardy-Weinberg equilibrium was applied. Comparison of the 17*β*-estradiol serum concentrations between the different genotype groups was performed with the use of the Kruskal-Wallis test.

In all cases, *P* < 0.05 was considered statistically significant.

## 3. Results

In the present study, 113 women with diagnosed mild or moderate depression (patients) and 219 women without depression (healthy controls) were genotyped for polymorphisms in six serotonergic candidate genes: *HTR2A*, *HTR1B*, *HTR2C*, *TPH1*, *TPH2*, and *MAO-A*. The data for allele frequencies and genotype distribution of these polymorphisms for the patients and the controls are presented in Table 1. Distribution of these polymorphisms was consistent with the Hardy-Weinberg equilibrium in the group with depressive disorders, as well as in the control group.

No significant differences were observed in the frequency of either the *5HTR2A* c.102C>T, *5HTR1B* c.861G>C, *HTR2C *c.68G>C,* TPH1 *218C>A, or *TPH2* c.1077A>G genotypes or alleles between the patients and the controls (Table 1).

The *MAO-A* c.1460C>T polymorphism in the patient group demonstrated a significant difference when compared to the control group. The frequency of the T allele in the group of women with depression was higher than that in the control group (*P* = 0.011) (Table 1). The frequency of the homozygous c.1460TT genotype in these groups reached 14% and 8%, respectively (Table 1). Although the frequency of the heterozygous c.1460CT was higher in women with depression (50%) than in women without depression (42%), this was not significant (Table 1).

We also undertook the test for association, using c.1460T as a risk allele (Table 1). The OR and CI were calculated for each genotype and compared with the homozygous values for the genotype of higher frequency among controls, which was set as the reference genotype. When the c.1460CC genotype was used as the reference, the combined c.1460CT + TT genotypes were associated with higher risk of depression (OR = 1.772; CI = 1.112–2.825, *P* = 0.0198).

17*β*-estradiol serum concentration was not associated with depression in postmenopausal women. No significant differences in 17*β*-estradiol serum concentration were observed between the healthy group of postmenopausal women without depression (*r* = 108.1 pg/mL), the mild depression (*r* = 102, 1 pg/mL), or the moderate depression (*r* = 74.2 pg/mL) patient subgroups.

Comparison of the 17*β*-estradiol serum concentration between patients with different genotypes of the *MAO-A* c.1460C>T polymorphism yielded a significant difference between the patients with wild-type or heterozygous genotypes (1460CC or 1460CT, resp.) and those with the homozygous genotype variant (1460TT) (Kruskall-Wallis, *χ* ^2^ = 11.960, *P* = 0.0025). In the subgroup of the homozygous 1460TT genotype, a significantly higher 17*β*- estradiol concentration was observed (*r* = 130.03 pg/mL) as compared to the 1460CT (*r* = 88.64 pg/mL; *P* = 0.0065) or 1460CC (*r* = 83.173 pg/mL; *P* = 0.0018) genotypes.

No significant correlations between plasma levels of 17*β*-estradiol and the other SNP genotype variants were observed.

## 4. Discussion

We found a significant contribution of the *MAO-A* c.1460C>T polymorphic variant to depression in postmenopausal women. An association between the *MAO-A* CT and TT genotypes and depression in postmenopausal women has been evidenced. A C-to-T substitution at the third base of codon470 in exon14 (Asp470Asp) of *MAO-A *results in *Eco*RV restriction length polymorphism. To date, significant associations have been observed between the T allele for the *MAO-A Eco*RV polymorphism and high MAOA activity, with levels ranging more than 50-fold among control subjects, as measured in cultured skin fibroblasts [28]. We provide evidence for an association between depression in postmenopausal women and the T allele of the *MAO-A* gene, previously linked to high MAOA enzymatic activity [26–28]. These findings are in agreement with the well-established action of MAOA inhibitors as antidepressants.

High-activity variants of the *MAO-A* gene have been also associated with OCD. This association, however, has been detected among OCD males with comorbid major depressive disorder, more likely having the high-activity T allele of the *MAO-A* gene than controls [23–25]. On the other hand, females with OCD were more frequently homozygous for the low-activity C allele of the *MAO-A Eco*RV variant compared to controls, with this allele also more frequent in female patients than in controls [26]. Considering that the *MAO-A* gene is localized to the X chromosome, our results may support a sexually dimorphic association between the gender groups as reported by others and the occurrence of alleles of the *MAO-A* gene polymorphism. These discrepancies, however, might also be due to the different populations that have been studied.

The fairly low relative risk of depression observed in postmenopausal women having the CT or TT genotypes (OR = 1.772) may be explained by a predisposition of patients in the postmenopausal period to depression by a variant that may not be in the *MAO-A *gene but within a closely linked, yet-to-be-discovered susceptibility gene. The most likely explanation, however, is that the linked, functional, *MAO-A* high-activity variant has low penetrance or imposes a risk on only a subset of postmenopausal women with depression. This theory is consistent with previous reports of a beneficial effect of MAOA inhibitors in certain OCD cases, although, in general, treatment of OCD with MAOA inhibitors does not seem to be as effective as treatment with selective serotonin reuptake inhibitors (SSRIs) [34]. An association between high-activity variants of the *MAO-A* gene and depression in postmenopausal women suggests, however, that some of them will also respond well to MAO inhibitors.

Estrogen likely promotes serotoninergic neurotransmission by influencing release, metabolism, reuptake, or synthesis [35]. It has been reported that women with a high-activity *MAO-A* genotype differ from men and from women with a low-activity genotype, which suggests that women with a high-activity *MAO-A* genotype may drive some previously reported sex differences in serotoninergic mechanisms [36]. Among women, but not among men, the concentration of the major serotonin metabolite, 5-hydroxyindoleacetic acid (Figure 1), was greater in those with a high-activity genotype than in those with a low-activity genotype [36]. For healthy women, plasma MAO activity is lowest at the time of ovulation, when 17*β*-estradiol production is greatest, but MAO activity increases during the luteal phase, when progesterone is secreted [30]. The estrogen deficiency of postmenopausal women who have not used HRT results in higher MAO activity. This may have an influence on the risk of depressive disorders in postmenopausal women [31–33]. Nevertheless, the genetic association between the *MAO-A Eco*RV polymorphism and depression has never been analyzed in postmenopausal women. Several lines of evidence support the hypothesis of an antidepressant effect of estrogens exerted via inhibition of the MAO pathway in women [37]. High-dose estrogen significantly decreased MAOA activity in the hypothalamus and amygdala in adult female rats, with no significant changes in MAOB activity in these areas of the brain [38].

In a human neuroblastoma cell line with transfected cDNA of the estrogen receptor (SK-ER3), estrogen receptor activation by a physiological concentration of 17*β*-estradiol was correlated with a marked decrease in MAOA activity [39]. We did not determine MAOA activity in the blood samples studied, and although concentrations of 5-HT and 17*β*-estradiol were measured, plasma levels of either 5-HT or 17*β*-estradiol did not differ between particular groups of postmenopausal women. However, the Kruskal-Wallis test revealed that the high-activity T allele of the* MAO-A Eco*RV polymorphism is markedly associated with a higher 17*β*-estradiol concentration, thus favoring the hypothesis of the presence of a functional link between estrogen and MAOA activity in human cells of neural origin. In fact, a reverse causality in the relationship between high 17*β*-estradiol levels and depression disorders may have a protective role in the homozygous 1460TT *MAO-A Eco*RV subgroup of the studied postmenopausal women. On the other hand, the concentration of 17*β*-estradiol may not be sufficient in preventing depression in postmenopausal women that are not taking HRT [40].

We concluded that the 1460T allele of *MAO-A* appeared with a significantly higher frequency in depressed female patients than in the control group, and the patients with the 1460CT + TT genotypes showed an increased risk of depression, which indicates that the 1460T allele of *MAO-A *may be a risk factor for depression in postmenopausal women.

## Figures and Tables

**Table 1:** The genotype distribution between postmenopausal women with (*w*) and without (*w*/*o*) depression.

Polymorphism	*n*	Genotype and allele distribution absolute number (frequency)					Allele *P* Value	Genotype Odds ratio (95% CI); *P* Value
		CC	CT	*TT*	C	T		
*5HTR2A *c. 102C>T (SNP 6313)	w depression	50	44	19	144	82	*P* = 0.735	0.769 (0.485–1.219)^a^; *P* = 0.2634^b^
	Total 113	(0.44)	(0.39)	(0.17)	(0.64)	(0.36)		
	w/o depression	83	107	29	273	165		
	Total 219	(0.38)	(0.49)	(0.13)	(0.62)	(0.38)		

		GG	GC	**C** **C**	G	C		
*5HTR1B *c.861G>C (SNP 6296)	w depression	62	44	7	168	58	*P* = 0.635	1.094 (0.693–1.728)^a^; *P* = 0.700^b^
	Total 113	(0.55)	(0.39)	(0.06)	(0.74)	(0.26)		
	w/o depression	125	83	11	333	105		
	Total 219	(0.57)	(0.38)	(0.05)	(0.76)	(0.24) )		

		GG	GC	**C** **C**	G	C		0.996 (0.610–1.628)^a^; *P* = 0.989^b^
*5HTR2C *c. 68G>C (SNP 6318)	w depression	78	30	5	186	40	*P* = 1.000	
	Total 113	(0.69)	(0.27)	(0.04)	(0.82)	(0.18)		
	w/o depression	151	57	11	359	79		
	Total 219	(0.69)	(0.26)	(0.05)	(0.82)	(0.18) )		

		CC	CT	*TT*	C	T		1.772 (1.112–2.825)^a^; *P* = 0.0198^∗b^
*MAO-A *c.1460C>T (SNP 1137070)	w depression	41	56	16	138	88	*P* = 0.011*	
	Total 113	(0.36)	(0.50)	(0.14)	(0.61)	(0.39)		
	w/o depression	110	91	18	311	127		
	Total 219	(0.50)	(0.42)	(0.08)	(0.71)	(0.29) )		

		CC	CA	*AA*	C	A		
*TPH1 *218C>A (SNP 1800532)	w depression	44	49	20	137	89	*P* = 0.801	1.014 (0.637–1.615)^a^; *P* = 0.9533^b^
	Total 113	(0.39)	(0.43)	(0.18)	(0.61)	(0.39)		
	w/o depression	86	99	34	271	167		
	Total 219	(0.39)	(0.45)	(0.16)	(0.62)	(0.38) )		

		GG	GA	*AA*	G	A		
* TPH2 *c.1077G>A (SNP 7305115)	w depression	45	51	17	141	85	*P* = 0.737	0.870 (0.546–1.386)^a^; *P* = 0.5573^b^
	Total 113	(0.40)	(0.45)	(0.15)	(0.62)	(0.38)		
	w/o depression	80	106	33	266	172		
	Total 219	(0.37)	(0.48)	(0.15)	(0.61)	(0.39)		

The women's groups were classified based on the severity of depression assessed by the Hamilton rating scale for depression: w/o depression (0–7), mild depression (8–17), and moderate depression (18–25). The 113 women with depression included 82 women with mild depression and 31 women with moderate depression.

^a^The Odds Ratio was calculated for patients homozygous or heterozygous carrying risk allele *vs*. homozygous.

^b^Fisher exact test was used for comparison of patients with depression versus patients without depression.
